# Dynamic Scheduling and Adaptive Power Control for LoRaWAN-Based Waste Management: An Energy-Efficient IoT Framework

**DOI:** 10.3390/s26030844

**Published:** 2026-01-27

**Authors:** Yongbo Wu, Cedrick B. Atse, Ping Tan, Xia Wang, Huoping Yi, Zhen Xu, Jin Ding, Priscillar Mapirat

**Affiliations:** School of Automation and Electrical Engineering, Zhejiang University of Science and Technology, Hangzhou 310023, China; wuyongbo@zust.edu.cn (Y.W.); 912107802014@zust.edu.cn (C.B.A.); 122126@zust.edu.cn (H.Y.); 123020@zust.edu.cn (Z.X.); 114084@zust.edu.cn (J.D.); 912307802011@zust.edu.cn (P.M.)

**Keywords:** IoT, LoRa, low power consumption, smart waste bin

## Abstract

**Highlights:**

**What are the main findings?**

- Dynamic scheduling combined with adaptive data rate and power control significantly reduces LoRa node energy consumption.
- The optimized system maintains reliable data transmission while extending battery lifetime.

**What are the implications of the main findings?**

- Reduce energy usage.
- Reduce overflow and operational costs in smart waste management systems.

**Abstract:**

Efficient waste management is a critical challenge in urban areas. This paper explores the optimization of power consumption in a smart bin management system using LoRa (long-range) communication technology. LoRa’s low-power, wide-area capabilities make it an ideal choice for IoT-based waste management systems. However, energy efficiency remains a crucial factor for ensuring the long-term sustainability of such systems, to avoid frequent intervention and reduce operating costs. This study employs advanced optimization techniques to minimize the energy usage of LoRa nodes while maintaining a reliable data transmission and system performance. By integrating a dynamic scheduling algorithm based on the usage of bins, and a custom adaptive data rate and power algorithm, the proposed solution significantly reduces the system’s energy impact. The performance of the system is evaluated through simulations and real-world deployment, where the results demonstrate a significant reduction in energy usage, over 84%, a longer battery life, and fewer maintenance interventions. The findings provide a scalable and energy-efficient framework for deploying smart waste management systems in resource-constrained environments.

## 1. Introduction

Efficient waste management is a growing concern in urban areas. Rapid urbanization and population growth have led to increased waste generation, often outpacing the capacity of traditional waste collection systems. Inefficient waste management not only leads to environmental pollution but also poses significant health risks to urban populations [1]. Addressing these challenges requires innovative, cost-effective, and scalable solutions that optimize resource utilization while enhancing system performance.

The application of Internet of Things (IoT) technologies in waste management has gained significant attention in recent years as cities seek intelligent solutions to cope with increasing waste generation and limited collection resources. Existing studies primarily focus on automation, monitoring, data intelligence, and system-level optimization to improve operational efficiency and service quality. García González et al. [2] propose an autonomous waste classification system based on multi-agent systems and blockchain technology. Their approach emphasizes decentralized intelligence and low-cost implementation to improve the waste sorting accuracy and system transparency. While the proposed system demonstrates effective automation and decision-making capabilities, its primary focus lies in waste classification rather than communication efficiency or energy consumption. Nevertheless, the work highlights the importance of intelligent system design and decentralized architectures, which are also relevant for large-scale smart waste management deployments. Security and privacy aspects of smart waste management systems are examined by Brighente et al. [3], who analyze these systems from a cyber–physical system perspective. The authors identify potential vulnerabilities in sensing, communication, and data processing layers, emphasizing the need for secure and reliable IoT infrastructures. Although energy efficiency is not directly addressed, the study underlines the importance of robust communication mechanisms, which indirectly influence system reliability and energy usage, particularly in low-power wireless networks such as LoRaWAN. Several studies focus on real-time monitoring of waste bins using sensor-based IoT solutions. Pires et al. [4] present an IoT-enabled system that employs Time-of-Flight (ToF) sensing technology to monitor urban garbage levels in real time. Their solution provides accurate fill-level detection and enables timely waste collection. However, the study does not consider adaptive energy-aware scheduling, which limits the long-term autonomy of battery-powered sensor nodes. Similarly, Okechukwu et al. [5] discuss the transformation of urban and municipal plastic waste management through smart technologies, highlighting the potential of IoT-driven monitoring and data analytics to improve sustainability. While the work provides a broad overview of smart waste technologies, energy efficiency and low-power communication optimization are not explored in depth. Machine learning techniques have also been integrated into smart waste management systems. Kalaiselvan et al. [6] propose an automated waste management system using Support Vector Machine (SVM) algorithms to classify waste and improve decision-making. While the inclusion of machine learning enhances system intelligence, the study does not address the energy constraints of IoT nodes or the optimization of wireless communication parameters, which are critical for long-term field deployment. Other works emphasize fault detection and system robustness in IoT-based waste management. Singh et al. [7] present an IoT-integrated smart waste management system with real-time monitoring and fault detection capabilities. Their approach improves system reliability and maintenance efficiency by enabling the early detection of abnormal conditions. However, the energy impact of continuous monitoring and communication is not explicitly analyzed, which may affect system autonomy. Similarly, Surya et al. [8] introduce the Amphi framework, a smart urban waste management solution designed for real-time monitoring and sustainable disposal. While the framework demonstrates the benefits of real-time data acquisition and integrated system design, it does not incorporate power control mechanisms to minimize energy consumption. IoT-assisted waste collection systems aimed at optimizing urban operations are further investigated by Khan et al. [9], who propose an efficient smart waste collection framework for urban smart cities. Their system focuses on optimizing waste collection routes and improving operational efficiency through sensor data and centralized decision-making. Although the framework demonstrates improvements in logistics and service quality, it assumes frequent data transmission and continuous system operation.

Using LoRa technology here reduces costs significantly because it allows long-range communication without the internet, which could introduce a huge expense [10,11,12]. By equipping waste bins with IoT sensors and using LoRa for communication, municipalities can monitor fill levels in real time, optimize collection routes, and especially, reduce operational costs. However, one of the primary challenges in deploying sensor-based devices is autonomy in energy. In this case, given the quantity of waste produced by citizens, it is important to build a system that can live as long as possible without constant technical intervention, to really achieve the enhancement desired. So, the longevity of the system depends on the battery life of IoT devices, which is directly affected by the communication protocols, data transmission frequency, and power engaged. In resource-constrained areas, optimizing energy consumption is crucial to ensure the long-term sustainability and affordability of smart bin management systems. We can draw on Khalifeh’s work [13], where the author deals with the optimization of the energy consumption level in a LoRaWAN network. They have developed an algorithm for the selection of the transmission power considering the receiver sensitivity and a reinforcement learning algorithm to select an appropriate spreading factor based on the path loss of the communication link between the node and the gateway. During the test, a transmission occurs each 4 min. The results show a significant reduction in the power consumption when comparing this to another paper, where another algorithm is used for the same purpose but where the transmission power is fixed at 14 dBm. Only the spreading factor changes. In this paper, the author focuses the energy-saving solution on the optimization of the communication link, and there is one transmission each 4 min. Whatever the optimization brings to the communication link, the current consumption is at least 10 mA, because apart from LoRa, the current consumption of the other components must be considered. Zhang [14] proposes an algorithm to reduce the number of packet collisions in order to minimize the energy consumption of the network. In fact, the data extraction rate is maximized, which ensures a smooth selection of SF and TP. Y.A. [15] worked on optimizing power allocation in LoRaWAN IoT applications, with a framework named Best Equal LoRa (BE-LoRa) to optimize the packet delivery ratio and energy efficiency. The framework or power allocation algorithm is mounted on the LoRaWAN server to replace the default ADR algorithm provider. The results show that BE-LoRa can enhance the delivery ratio by 17.44% and reduce power consumption by 46% compared to LoRaWAN ADR. Paper [16] proposes energy-efficiency LoRaWAN-based wireless sensors and a multi-agent reinforcement learning (MARL) algorithm to improve the energy efficiency of the sensors. This is specifically for a dynamic underground environment. The paper notes the limit of wireless underground sensors provided by the recent integration of LoRaWAN-grade massive machine-type (MTC) technology, which proves its insufficiencies: limited battery energy and massive data collision from different underground environments. The MARL algorithm serves to enhance energy efficiency, considering the link quality, energy consumption, and packet collisions. The results show that the proposed solution can quickly optimize the energy efficiency better than the traditional LoRaWAN ADR algorithm [17,18,19,20], since it was tested at different underground environment and network scales. Like the first paper, these are also focused on the reliability of communication. That is important, which is why in this paper, one strategy addresses the reliability of communication. But the particularity of the system is to adjust the sleep time according to the frequency of use of the SWB, a strategy to improve the energy efficiency. Therefore, in addition to treating the reliability of the communication according to the adaptive data rate and power strategy, the energy efficiency of the system is also boosted by the Dynamic Scheduling Algorithm, which gives the system the appropriate sleep time, to avoid it working when not necessary, thus saving the battery life. This paper aims to address the energy efficiency challenge by proposing optimization techniques for LoRa-based smart bin management systems. In this study, two strategies are developed and combined to minimize energy usage without compromising the communication reliability and system performance. The first strategy is the sleep time dynamic scheduling algorithm, based on the usage of the bins. Putting the system in the sleep mode is an efficient way to preserve the battery life of the system, but in this case, it is also important to consider the frequency of usage of the bins. The main contribution of this paper is the DSA + ADRPA technique, that is, the first technique aimed at reducing the power consumption of the system while avoiding an overflowed waste bin. The second contribution is an energy-efficient system, while guaranteeing a reliable data transmission. This consists of a custom adaptive data rate and power algorithm, to assign convenient parameters automatically, which allows the system to be energy efficient even when the environment changes.

This paper is structured as follows: In Section 2, the system is described, including the different parts, and the system’s operation is detailed. In Section 3, the solution proposed by this paper is explained. Section 4 presents the experiment procedure and the tests performed, while Section 5 discusses the results obtained. Some concluding remarks and future works are highlighted in Section 5.

## 2. The System

**Figure 1:**
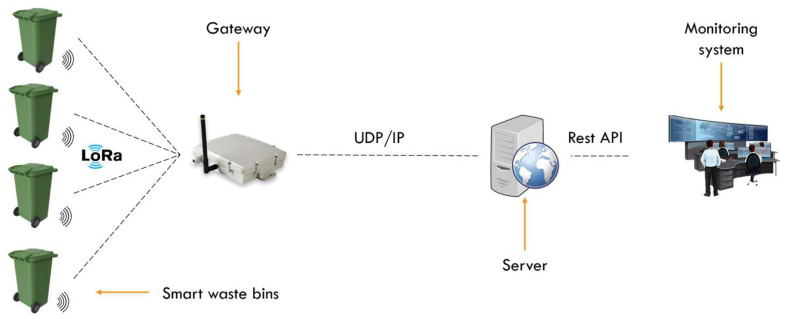
Architecture of smart waste bin management system.

The system consists of smart waste bins that measure the waste level using sensors and transmit the data via LoRa to the gateway, and then, through the gateway, the data is transmitted over the internet to be monitored by users, with a platform that helps them to view and control the system. The system contains three different parts, as shown in Figure 1. The first part is the smart waste bin (SWB), which is in charge of gathering data, in this case, the waste level, and sending it to the gateway. The second part is the gateway, which centralizes all data from the SWB and sends them to the control system. The gateway is the bridge between two different networks, LoRa and the internet, which allows the SWB to communicate with the control system. And the last part is the control system, which is a web platform that monitors and manages the data derived from the SWB via the gateway. This is the general architecture of IoT systems, which consist of a physical or sensing layer (smart waste bin), also called the IoT end device, a network and data processing layer (LoRa, gateway), also called the IoT gateway, and the application layer (control system). IoT devices are mostly battery-powered, which means they are installable anywhere. So, when we talk about battery-powered devices, the battery life is automatically addressed because without a battery life, we cannot talk about autonomous devices. So, since the purpose of this paper is to address energy consumption optimization, all studies will be focused on the SWB.

**Figure 2:**
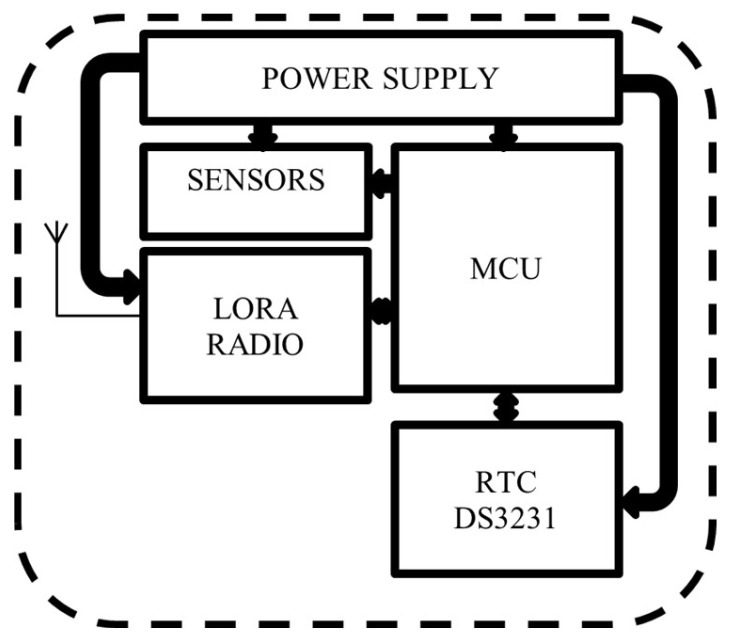
Smart waste bin architecture.

**Figure 3:**
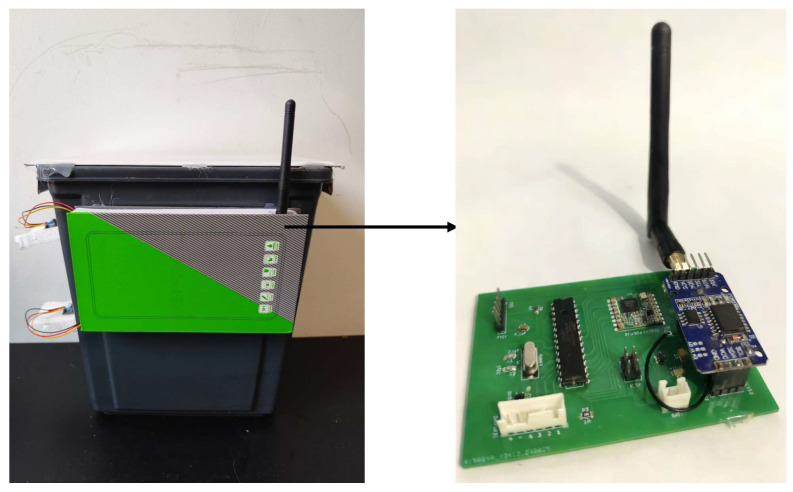
Smart waste bin circuit board.

**Figure 4:**
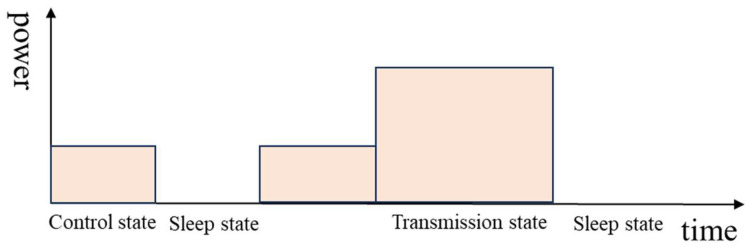
System operation.

The SWB consist of a power supply, sensors, LoRa radio, MCU, and RTC DS3231 (Figure 2). The MCU’s function is managed by the ATMEGA328P, which is an 8-bit AVR microcontroller. It integrates low-power modes, making it well suited for energy-constrained IoT and sensor-based applications. Sensors are located in two different parts of the SWB: one sensor on the cover, and two sensors on the body for medium- and high-level detection. The sensor used here is KY-32 for its accuracy and its capability of working well under light [21], because the IR sensor value is influenced by light, but this system should work under light. The SWB is also equipped with a LoRa radio module for data transmission. RFM98 is the LoRa module used for data transmission. It matches with all requirements, and it is energy efficient. The whole system is powered by a 5 V/3000 mAh battery. Figure 3 shows the components on the board. The SWB checks the content level at each defined time, and when a medium or high level is reached, the information is transmitted through the LoRa module to warn the municipalities. The SWB has different working states: the sleep state, control state, and transmission state, and the SWB is accessible at any time by users. Figure 4 illustrates the working of the system over time and how the states alternate. When the system does not operate, it is in rest (the sleep state), which is represented by the line. After an amount of time, it changes to the control state to check its content level, and from here, it can switch back to the sleep state or enter the transmission state, which depends on the value reported by the sensors. If the sensors report nothing, neither the medium level nor the high level, it switches to the sleep state again. But if the sensors report one of these levels for the first time, then the system switches to the transmission state to update the state of the SWB for the municipalities.

The system cannot switch directly from the sleep state to the transmission state. The control state is the center; it is the normal state of the system, where all the system components are on. To reinforce energy optimization, a specific function has been added to the hardware. The function is a switch function (on/off) using a transistor MOSFET mounted as a switch, and controlled by the MCU in the control state. For instance, in the sleep state, nothing occurs because the system sleeps. But sensors and the LoRa module still consume energy because they are constantly powered, and the energy consumed at this point is not insignificant. So, the role of the function is to power them off when they are not used in the process. As seen before, the system starts in the control state, which serves to check the content level. But before checking this, the sensors are disconnected from the power supply and also the LoRa module. So, the system powers and sets up the sensors to check the level and save the value, and after that, it disconnects the sensors again from the power supply. And if any specific level is detected, the system turns on the LoRa module, sets it up, and transmits data, and then it turns it off after a complete transmission, before going into the sleep state.

## 3. Low-Consumption Strategies

Most LoRa-based devices are battery-powered [22] in order to have an autonomous system and reduce costs. It is important to think about the battery life of such a system. Taking care to consider the longevity of the system in our case is very important. Considering the frequency of use of a waste bin, the system should have an adaptive working life to meet all needs in municipalities, and for that, a certain amount of autonomy is required for the system. To reduce power consumption, two strategies were implemented.

### 3.1. Dynamic Scheduling Algorithm

This strategy consists of adjusting the sleep time of the system relative to the frequency of waste bin usage. The sleep mode is the current energy-saving technique, and the common use of this strategy is to put the system to sleep for a fixed amount of time. Here, this technique will be applied beyond its common use, to suit this project [23]. In fact, the frequency of use varies at different times in the day. Life experience shows that waste bins are not used at the same frequency throughout a day. For instance, from 0 h to 5 h, waste bins are hardly used because it is bedtime. Accordingly, during this interval of time, the sleep delay of the system should arrange to have the system sleep for a long time. So, depending on the time in the day, the sleep delay is adjusted to match the use flow and save as much energy as possible.

Dynamic scheduling deals with different setting times, or, in other words, deals with adjustable times based on the frequency of use of the SWB. Here, the dynamic scheduling algorithm will set a sleep delay depending on the specific time in a day. In fact, for each period of time, a time value has to be defined. Based on daily life experience, a day can be subdivided into five intervals of time: interval 1 (0–5 h), interval 2 (5–8 h), interval 3 (8–17 h), interval 4 (17–20 h), and interval 5 (20–24 h), as the frequency of use of the waste bin is different for each interval. To do so, the system will register each opening time using a KY-032 infrared sensor, to detect when the cover is open, and the RTC module, to pick the time at which the cover is open. For each interval, the total opening duration $D_{i}$ is shown by (1):

(1) $$D_{i}=\sum_{J=1}^{N_{i}}X_{j}$$

where i represents the interval number (here, i=1, 2, 3, 4, 5), $X_{j}$ is the duration between two consecutive openings, and $N_{i}$ is the total opening times in each interval.

The sleep delay ($T_{i}$) of an interval is defined as

(2) $$T_{i}=\frac{\;D_{i}}{N_{i}},$$

Using (2), when we determine the sleep delay ($T_{i}$) in each interval, the periodic dormancy can be taken to save energy.

It is important to note that putting the system to sleep means putting the MCU only to sleep, so in order to reduce the current consumption in this strategy significantly, all unnecessary functions are disabled when the system is asleep. And this is achieved by the MOSFET transistor introduced before. The system is put in a deep sleep mode, because practically nothing occurs or is needed during the sleep mode, except for the RTC (Real-Time Clock) module, which is not controlled by the MCU, other than to set it up. Talking about the RTC module, it is the main component that allows the dynamic of this strategy. The RTC is a module that provides real-time value to the system. That is very beneficial, and it is exactly what the system needs, while the system’s operating schedule refers to the real times in a day (6 a.m., 5 p.m., …). The RTC module has its own battery, which will keep the time up to date even if the module is not powered. Then, before going into the sleep mode, the system schedules the wake-up time of the MCU by setting an alarm using the function above on the RTC. Since the RTC can work alone, it saves running the alarm scheduled; the MCU sets and activates the alarm before going to sleep. So, when the scheduled time is reached, a signal is generated from the SQ pin to wake up the MCU by using the interrupt pin, and then the routine restarts.

To sum up, the heart of the strategy is the sleep delay allocation algorithm, which gives all the dynamism of the system. Before entering the sleep mode, after checking, the MCU closes all functions (sensors and LoRa radio) and schedules and activates an alarm on the RTC module using the value given by the DSA. Once the time is reached, the MCU is woken up and restarts the process.

This strategy brings energy saving, but that is not enough. Beyond that, it also important to address all variables in the active mode in order extend the battery life for longer. The LoRa transceiver is the part to consider, because it has different operating phases, which can be moderated to avoid a huge current consumption. Accordingly, that is the main subject of the second strategy.

### 3.2. Adaptive Data Rate and Power Algorithm

This part of the paper concerns data transmission. That occurs in the active mode, and it is used by the MCU to transmit the sensor’s data. The transmission of data is achieved through different steps, and each step takes part in energy consumption. The transmission is sent using LoRa technology.

LoRa is an RF modulation technology for low-power, wide-area networks, created by Semtech [24]. Essentially, LoRa is a radio modulation technique derived from chirp spread spectrum (CSS) technology [25]. A crucial characteristic of the LoRa-based solution is the ultra-low-power requirement, which allows for the creation of battery-operated devices that can last for up to 10 years [24]. The modulation has some parameters that define how the signal will be generated. In fact, this strategy involves managing these parameters to give a reliable communication, while promoting low consumption. These parameters are the bandwidth (BW), spreading factor (SF), and coding rate (CR). The LoRa bandwidth defines the maximum information that can be transmitted per unit of time. Its unit is Hertz (Hz). The commonly used bandwidths are 125 kHz, 250 kHz, and 500 kHz. These bandwidths are outlined in the specifications of the LoRa chip used. To achieve long-range transmission, it is recommended to use a lower bandwidth. LoRa spreading controls the data transmission speed and chirp rate. It impacts the range, time on air, battery life, and sensitivity received [26]. Six spreading factors are commonly used: SF7 to SF12. The LoRa coding rate is made up of redundancy bits that allow the LoRa signal to endure short interference. The coding rate can be adjusted according to the degree of interference in the channel. Its value varies from 4/5 to 4/8.

**Figure 5:**
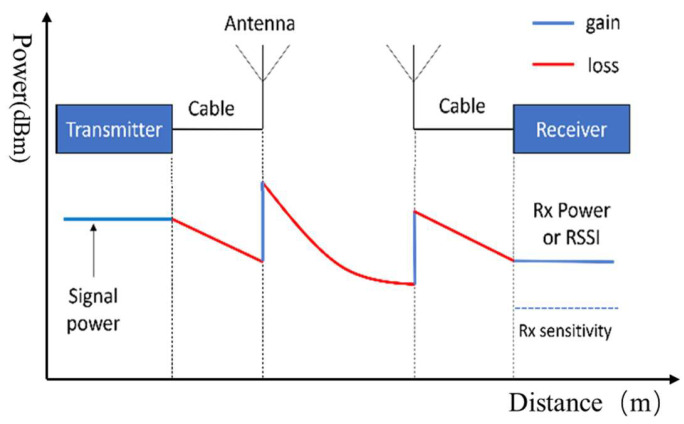
LoRa transmission signal power chart.

As you can see, to perform a LoRa transmission, these parameters need to be combined to ensure a good and reliable transmission. The importance of applying this strategy is that the environment in which the devices operate could change. Of course, the environment impacts the LoRa signal, because obstacles, like trees and buildings, decrease the strength of the signal. So, if the configuration is fixed in the device, it can work well, but when the environment changes, it will not work very well, and packet loss will be significant because the signal will be weak. To fix that, some parameters need to be adjusted to raise the strength of the signal. To automatically adjust the parameters, some data should be considered. The signal-to-noise ratio (SNR) is a metric that indicates the quality of the received signal relative to the noise. It determines whether the transmitted signal can be successfully decoded by the receiver. LoRa can operate with an SNR value from −20 dB to +10 dB. An SNR near +10 dB indicates that the signal has less interference. The Received Signal Strength Indication (RSSI) is a measure of the total power of the received signal. It contains the signal received and noise, and it indicates how strong the signal is at the receiver. It helps to determine the quality and reliability of the communication. And the sensitivity is the minimum RSSI required for successful communication. Its value depends on the receiver. Figure 5 shows the gain and loss of the signal power as its transmission progresses from the transmitter to the receiver.

This strategy involves adjusting the data rate or the spreading factor and transmission power (TP). These two factors allow us to adjust the range and the sensitivity of the signal (SF) and the strength of signal (TP). The spreading factor and transmission power affect the battery, but they are related. So, for an optimal situation, we have to have an optimal value of the TP, and on the other hand, an optimal value of the SF. Regarding the spreading factor (SF) and the transmission power (TP), the radio parameters used for LoRa transmissions are the bandwidth (BW) and the coding rate (CR). The bandwidths that are commonly used for LoRa are 125 kHz, 250 kHz, and 500 kHz. This value is commonly chosen based on the payload size. In this project, the lowest value (125 kHz) is chosen because the payload contains only 2 bytes (1 byte for device ID and 1 byte for the waste level). The value of CR used is 4/5. These values (BW and CR) are constant overall. Before running the algorithm, let us define some limits and constraints according to the capability of the RFM98 module:

- Receiver sensitivity: −148 dBm;
- SNR limit: −20 dB to +10 dB;
- Maximum Tx power: +20 dBm;
- Spreading factor: 7 to 12;
- RSSI average: −90 dBm.

Based on these values, the algorithm could adjust the SF and TP, according to the RSSI and SNR of the last signal received, for the next transmission.

To start, the LoRa module will be configured with the maximum value of each parameter, in order to ensure a first successful transmission. Based on the quality of the signal, the algorithm will adjust the parameters to find the appropriate levels. If the quality of the signal is very low, an error is reported on the management platform.

**Figure 6:**
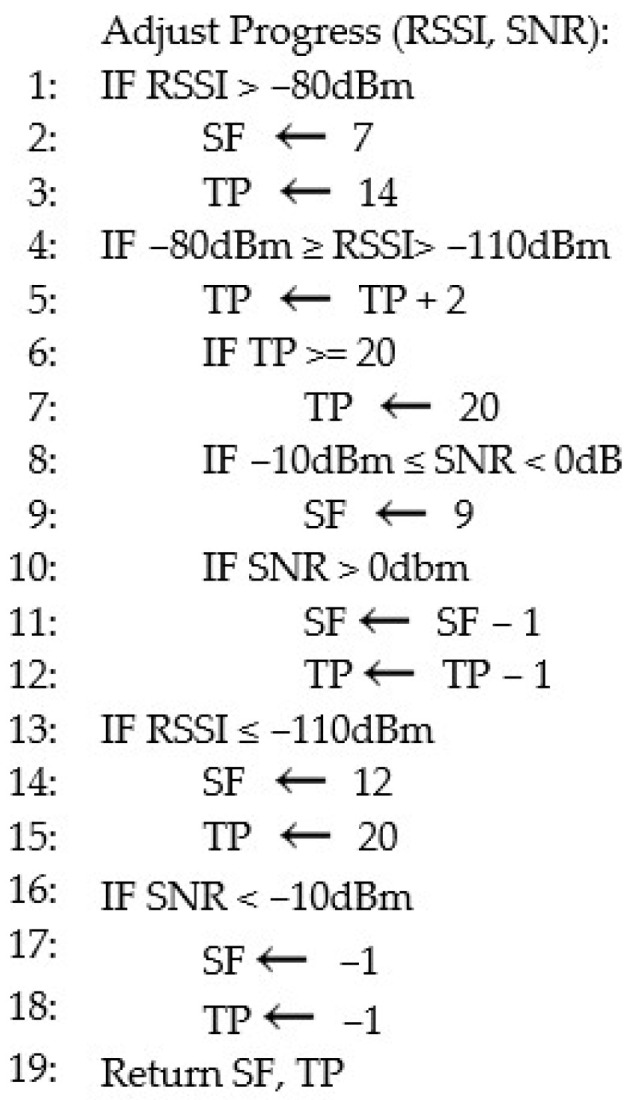
ADRPA algorithm.

To build the rule for parameter adjustment, the signal has been analyzed by different points of transmission, as shown in Table 1. The value obtained by this experiment was taken to build the adaptive data rate and power algorithm (Figure 6) for the system to dynamically adjust the transmission parameter to ensure successful and energy-efficient transmission. This is a test conducted to determine which parameter is the best considering the distance from the gateway. The test shows that within 100 m, the system could achieve a transmission using the lowest transmission power (14 dBm) and the lowest spreading factor (SF7). Above 100 m, the RSSI decreases (<−80 dBm), and some packet loss is noted with SF7 and TP 14. But a transmission could be sent with the adjustment of the spreading factor (SF9 to SF11) and the transmission power. SF9 is only selected because the result is almost the same when using SF10, and because SF9 is lower than SF10, since the system’s goal is energy saving. This adjustment is also made regarding the value of the SNR, which, depending on the obstacle, could decrease and make the signal weak (especially when the SNR is under zero). So, the spreading factor is adjusted, as it has more impact on the SNR. Over 250 m, the signal needs more strength to arrive, because the RSSI and SNR strongly decrease. So, the biggest value of the SF and TP is set. Considering these statements, wherever the device is used within the area of operation, the system could dynamically adjust the transmission parameters for the device to send correct information. When the value of the RSSI and especially the SNR is very poor, the value −1 is sent to signify an error from the system, which means that the device has lost communication with the gateway.

Standard ADR adapts slowly due to its dependence on multiple uplink frames and downlink commands, meaning it is less effective in environments where signal conditions change rapidly due to obstacles or distance variation. ADRPA is more responsive, as it adjusts parameters immediately based on the most recent RSSI and SNR measurements. The responsiveness of ADRPA is also due to the fact that the algorithm has been studied in a precise size of area. This helps the system to quickly choose the right parameters. This makes it particularly suitable for urban smart waste management deployments, where bins may be placed at varying distances and experience fluctuating channel conditions. Moreover, the algorithm’s ability to detect and report communication loss provides an additional layer of robustness not present in standard ADR.

## 4. Experiment

### 4.1. Deployment of the System

The deployment phase represents the final step of the system’s development, where the prototype is tested under real environmental conditions to validate its functionality, reliability, and efficiency. This stage aims to ensure that the smart waste bin system, designed with a low power consumption and long-range communication capabilities, can effectively operate in a real-world setting while maintaining a stable data transmission and monitoring performance.

The system was deployed in a controlled outdoor environment representative of typical waste collection areas, such as public spaces. The prototype, which consists of a plastic waste bin equipped with electronic components, was installed in an accessible location for regular users to interact with. Each smart waste bin (SWB) contained a microcontroller unit, two infrared sensors for fill-level detection, a LoRa transceiver module for communication, and a power supply unit.

The LoRa gateway, based on the M-GW1302s concentrator connector to a Raspberry Pi, was installed at a central point with a clear line of sight to the smart bins. The gateway was configured to receive data from multiple nodes (bins) simultaneously and forward the collected data to the central server through the internet. The backend system, hosted on a remote server, was responsible for storing, processing, and visualizing the collected data via a web-based dashboard.

**Figure 7:**
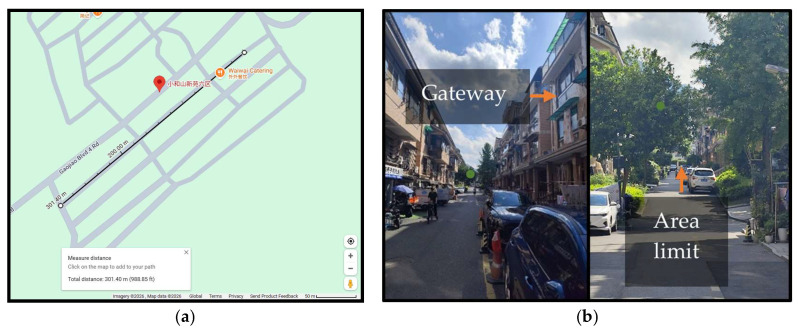
(**a**) GPS view of the test area; (**b**) real view of the test area.

To ensure stable communication, the deployment considered parameters such as the gateway height, antenna gain, and orientation. Each node was powered by a 5 V, 3000 mAh battery, chosen for its balance between size, capacity, and portability. The field test area (Figure 7) was approximately 300 m in radius.

### 4.2. Experimental Procedure

The experimental procedure was designed to evaluate the performance, reliability, and energy efficiency of the proposed smart waste bin system. This phase aimed to validate the two power-saving strategies under real operating conditions, ensuring that the system performs efficiently in both data acquisition and communication processes. The experiment was conducted in a controlled environment that simulates real-life usage scenarios, allowing users to interact naturally with the smart waste bin. The setup consisted of the complete system architecture, including smart waste bin prototypes, a LoRa-based communication network, and a centralized data monitoring platform.

#### 4.2.1. Preparation and Setup

**Figure 8:**
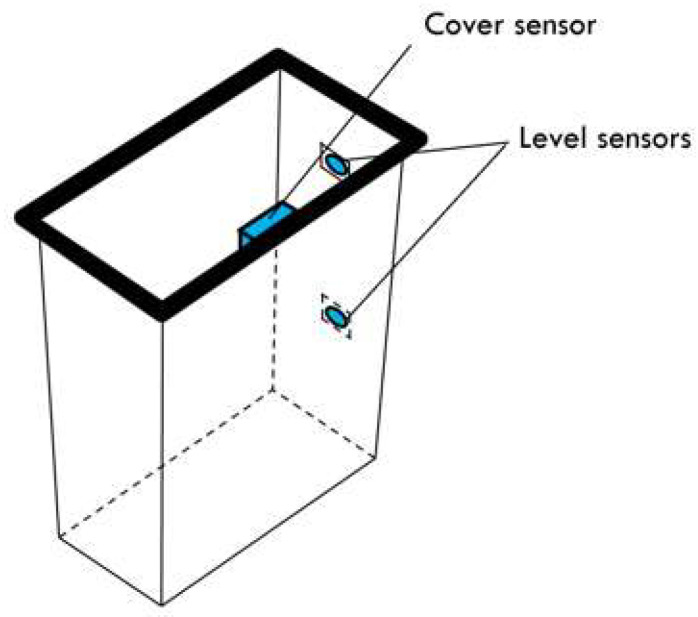
Positions of sensors.

Before experimentation, all system components were assembled, configured, and calibrated. The experimental setup involved the following steps:

The smart bin prototype was built using a plastic container, equipped with two infrared (IR) sensors placed at different heights, on at the middle to detect medium-level waste and another at the top to detect high-level waste (full bin condition). A third IR sensor was positioned under the lid to detect every opening and closing action, representing each usage of the bin (Figure 8). All sensors, microcontrollers, and communication modules were connected within a sealed electronic enclosure mounted on the back of the bin.

#### 4.2.2. Using Frequency Acquisition

This phase involves determining the optimal time over which the system will sleep, while ensuring good management. For that, every action on the SWB should be monitored (Table 2); the action here is when a person opens the lid of the SWB, while each opening is considered as a use. Recording these actions should allow for calculating the average frequency of use, in order to define an appropriate sleep duration. To track the real frequency in terms of time, the SWB was utilized by users, running a special program, and a sensor was mounted on the top of the bin (Figure 8). When the lid is closed, the sensor is active, and when the lid is lifted up, the sensor is inactive and an action is registered.

**Figure 9:**
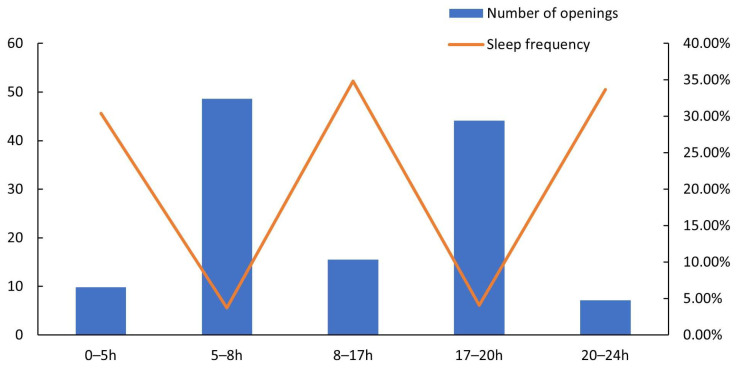
Sleep frequency of smart waste bin.

The expected result is to derive the average time in minutes between each action involving the SWB. The time value obtained for each interval will be the sleep delay of the SWB. Table 2 and the chart in Figure 9 show the number of times the cover of the SWB was opened and the sleep frequency of each interval. The data were collected over two consecutive days. A sample of 250 interactions was used, averaging 125 interactions per day.

The frequency is the time that the SWB should sleep for, obtained using Table 2. Based on the results obtained, it was noted that in certain periods of the day, the frequency of use differs. Based on that, the data were subdivided into five groups, as illustrated in Table 3. From 00 to 05 h, 08 to 17 h, and 20 to 24 h, the sleep duration is longer than from 05 to 08 h and 17 to 20 h, which means that during the three first intervals, there was less interaction than during the last two intervals.

For instance, from 0 to 5 h, the SWB should sleep and wake up each 30 min 4 s, and from 5 h to 8 h, each 3 min 7 s. The values obtained in this phase will be used for the normal working of the SWB. Because this step concerned learning the behavior of users, the system was required to stay alive for the whole test time. Then, the system was switched to its normal working mode using the scheduling time obtained for the sleep mode. The impact of the DSA was analyzed by measuring the current consumption in both the active and sleep states, using an amperemeter connected in series with the power supply.

#### 4.2.3. Evaluation of the Adaptive Data Rate and Power Algorithm

This experiment focused on optimizing LoRa communication energy by dynamically adjusting the spreading factor (SF) and transmission power (TP) according to the received signal quality. Five measurement points were established at increasing distances from the gateway. Table 4 presents the condition of the test path.

At each point, 10 transmissions were made every 10 s. The gateway recorded the RSSI and SNR. The node adapted the SF and TP values based on ADRPA conditions:

- Good signal (RSSI > −80 dBm), use SF7–SF8 and TP = 14 dBm;
- Moderate signal (−110 dBm < RSSI ≤ −80 dBm), use SF9–SF10 and TP = 17 dBm;
- Weak signal (RSSI ≤ −110 dBm), use SF11–SF12 and TP = 20 dBm.

The LoRa gateway supported uplink metadata processing, including the RSSI and SNR, which were used to inform data rate adjustments. This strategy aims to reduce the transmission time and power usage by adjusting the communication parameters considering the quality of the signal. The maximum distance in the test was 300 m away from the gateway. In order to check the reliability of the ADR algorithm, data were collected at different points, where each point represents an SWB at a different distance from the gateway. In this way, the ADR should show how it adjusts parameters for each point to ensure a good transmission while reducing the transmission time.

For the points closer to the gateway (within 0 to 100 m), the node was adapted to higher data rates (SF7–SF8), the power was reduced to 14 dBm, and a shorter transmission time was noted (around 45–60 ms). However, for the other points with minor obstructions, like trees and parked vehicles, the node was adapted between SF9 and SF12, and an increase was noted in the transmission time (from 90 up to 120 ms).

## 5. Results and Discussion

The results show how the system can adapt to the frequency of use of the waste bin, while keeping energy consumption to a minimum. The frequency of use of the smart waste bin varies during the day. So, during some moments in the day, the SWB is not used for some hours. During these moments, the sleep time of the SWB gives a strong opportunity for the system to enter a low-power standby mode without compromising data accuracy or responsiveness. The results of the test have shown that the system can schedule deep sleep modes during low-interaction periods to conserve energy. With an understanding of how and when users interacted with the bin, the smart bin could better balance resource efficiency, responsiveness, and longevity. In most applications, a static sleep time is used. This could help with saving energy, but regardless of responsiveness, which can lead to overflowing waste. Compared to other strategies, the strategy applied here is much better. As the result of the first strategy is to have dynamic sleep times, based on experience, Table 1 gives the average time between interactions. This means, for example, that between 0 h and 5 h, the SWB is unused for 30.36 min at least nine times. During this time, the SWB system can sleep because it is not used. In this way, the time between interactions ascertained in Table 1 is automatically considered as dynamic sleep times. To really determine the impact of this strategy on the power consumption, the real value of the current consumption has to be known. To find that value, the duration of a transmission cycle has to be determined. Considering that a transmission cycle combines the sleep and active modes, the durations of the sleep mode and the active mode have to be determined, and as a day is subdivided into five intervals, the sleep and active modes’ durations in each interval must be found as well. The transmission cycle time of an interval (${TTC}_{i}$) is determined with (3):

(3) $${TTC}_{i}=t_{active\_i}+t_{sleep\_i},$$

The value of ${NC}_{i}$ (number of transmission cycles of an interval) is given by Table 2, and this value is determined with (4):

(4) $${NC}_{i}=\frac{d_{i}}{\left(t_{active\_i}+t_{sleep\_i}\right)},$$

where $t_{active\_i}$ and $t_{sleep\_i}$ are, respectively, the active mode duration and sleep mode duration of an interval, and $d_{i}$ is the interval duration. In Table 5, the cycle duration is the result of ($t_{active}+\;t_{sleep}$). So, the number of cycles is simply ascertained by dividing the total duration ($d_{i}$) by the cycle duration.

In this way, the values of $T_{active\_i}$ (total active time) and $T_{sleep\_i}$ (total sleep time) for an interval can be calculated using (5), as shown in Table 6.

(5) $$\{\begin{array}{c} T_{active\_i}=t_{active\_i}\cdot {\;NC}_{i}\\ T_{sleep\_i}=t_{sleep\_i}\cdot {NC}_{i} \end{array}$$

Mode means active or sleep. The next step is to determine the current consumption of the SWB in a day, using the previous values.

To find the daily current consumed by the SWB, the total operating time (daily active time and sleep time) and the operation current have to be considered. The daily current consumed by the SWB is given by (6):

(6) $$I_{daily}=I_{active}+I_{sleep}$$

$I_{active}$ or $I_{sleep}$ is the current consumed during the active or the sleep mode, which is defined as the total daily active ($T_{active}$) or sleep time ($T_{sleep}$) multiplied by the total current consumed in one day, as shown by (7):

(7) $$I_{total}=T_{active}\cdot I_{active}+T_{sleep}\cdot I_{sleep}$$

During the sleep mode, the device consumes 1.3 mA, and in the active mode, around 27.5 mA. These values were noted using a multimeter during the test. In a day, the device is active for 20.217 min, which corresponds to 0.337 h, and it sleeps for 1419.793 min ≈ 23.663 h. This results in a daily current consumption of 40.03 mAh, and then an average current of 1.668 mA. The normal operation of the device consists of an active mode and an idle mode. In the idle mode, the sensors and LoRa radio are not used but still active. The current consumption spent in the active mode is still 27.5 mA, and it is around 10 mA in the idle mode, which results in a daily consumption of 245.897 mAh and an average current of 10.245 mA. This analysis (Table 7) shows that the strategies developed in this project are able to extend the battery life by reducing energy consumption by up to 80%.

On the other hand, the integration of the ADR played a significant role in optimizing both the communication performance and energy consumption of the SWB. The results obtained during the test show how the ADR balance improves the reliability and efficiency in data transmission by dynamically adjusting the data rate and transmission power. The ADR principally and directly impacts the time on air, which is a crucial thing to consider for energy saving.

**Figure 10:**
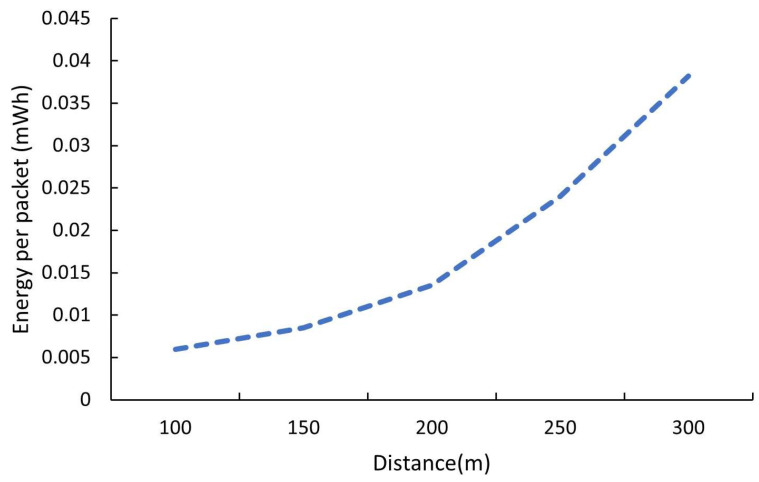
Energy per packet vs. transmission distance.

The energy per packet increases exponentially with the distance (as shown in Figure 10), mainly due to higher spreading factors (SFs) and transmission powers (Table 8). Between 100 m and 300 m, the energy cost increases by more than six times, showing the direct influence of adaptive data rate control. The energy per packet value is obtained by (8), where *E* is the energy in a milliwatt-hours (mWh), *V* is the supple voltage (5 V), *I* is the average current in amperes (A), and *t* is the transmission duration in seconds (s).

Considering that 1 watt-hour = 3600 joules, so

(8) $$E(mWh)=\frac{E\left(J\right)\times 1000}{3600},$$

Substitute *E*(*J*) from (8) to produce (9):

(9) $$E(mWh)=\frac{V\;\times I\;\times t\;\times 1000}{3600},$$

The final expression gives (10):

(10) E(mWh)=V(v)×I(A)×t(s)×0.2778,

Based on this result, the daily transmission energy using ADRPA can be determined. Considering that the number of transmissions per day from Table 5 is 121.29 (sum of the *NC_i_* values), the daily transmission energy is computed by (10), and Table 9 shows the daily energy per distance. $E_{per\_tx}$ is the energy per transmission, and *N* is the number of transmissions per day (121.29).

(11) Daily energy(mWh)=×N,

Convert the daily energy to mAh (at 5 V) using (12):

(12) $$Daily\;(mAh)=\frac{Daily\;energy\left(mWh\right)}{5},$$

ADRPA’s per-day cost for transmissions is very small, because each transmission is short. The further the node, the larger the per-tx energy and the larger the daily consumption. By using the static parameter (SF12/TP20) per transmission, and considering that the measured current for the maximum power is around 134 mA and the transmission duration is 0.4 s, the static energy per transmission (mWh) is 0.07445 mWh. This gives a daily static transmission of 0.07445 × 121.29 = 9.03 mWh, so 1.8 mAh/day; this result is obtained in Table 10.

**Figure 11:**
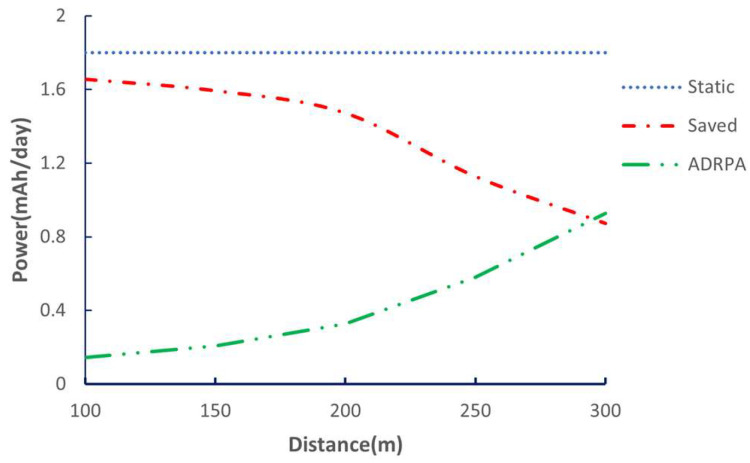
ADRPA vs. static and daily energy saved.

ADRPA saves roughly 0.8 to 1.6 mAh per day compared to always using a conservative static configuration. There is more saving for closer nodes, as can be seen by consulting Figure 11.

When combining the two strategies, a significant impact is noted on the power consumption of the system. Table 11 provides the total daily consumption of the system. And the new daily consumption is expressed by (13):

(13) $$New\;daily\left(mAh\right)=DSA_{total}-ADRPA_{saved},$$

Considering that the battery capacity is 3000 mAh, the battery life value is calculated using (14):

(14) Battery life (Days)=3000 mAh/daily (mAh),

The DSA already provides a very large energy reduction. ADRPA then adds a further 5 to 8% improvement in battery life depending on the distance. Combining both produces large- and fine-grained optimizations, as the DSA reduces the baseline energy massively, and ADRPA tunes the remaining transmission cost, which pushes the system toward maximum autonomy by saving energy over 84%.

Without the ADR, a transmission could remain incomplete, and that could sustain over a long period. The spreading factor and the consumption cannot be managed. That means that when using a Tx power of 20 dBm and SF12, the transmission drains a constant and huge current based on the setting, and while the transmission lasts, power is consumed. So, without the ADR, the first strategy of this paper could remain insufficient, considering that the SWB waits for a completed transmission before going to sleep. That leads us to say that the two strategies are a perfect match, as they significantly reduce the power consumption. Azahra [27] has not really applied the ADR in his paper, but has carried out different tests including the RSSI and transmission delay tests. His goal for these tests is to determine the maximum distance for the system to operate well. The deployed devices are set with the same static transmission parameters. In this way, the risk of a bad communication can be significant because when the environment changes, obstacles could appear, and in this case, the parameters that have been set reach their limit. The condition for tuning parameters in this paper is based on an initial experiment. In the same way as the previously cited paper, tests of the RSSI have been performed, and also the SNR with different transmission points, which represent the distance from the gateway. During these tests, the limit of some set of parameters has been noted, considering the distance of the node from the gateway and the potential for an obstacle in the field of transmission. After noting the set of parameters that can ensure a good transmission considering the distance from the gateway, Figure 6 has been made to dynamically change the SWB transmission parameters. In this way, the risk of a bad communication is weakened compared to the previous papers’ strategies, because the SWB can adapt to the environmental conditions.

From the experimental results, it can be noted that the combination of the two strategies brings significant fresh air to waste management solutions, facilitating disposal via SWBs an in operating area. This is achieved by addressing the amount and flux of waste production, while watching over the battery life.

## 6. Conclusions

This paper addresses the optimization of the power consumption in a smart waste bin management system, to extend the battery life of smart waste bins, which is important for such systems. Since the purpose of these systems is to enhance waste management, it is useful for smart waste bins to have long battery lives for a most efficient, helpful, and autonomous solution. This study optimizes the power consumption of the SWB system by integrating an adaptive data rate and power algorithm and a dynamic scheduling algorithm. The optimized system extended the battery life while maintaining reliable communication, and it connected the behavior of users to the SWB. Together, the DSA and ADRPA create a multi-dimensional low-power strategy, which extends the battery life by around 84%, reduces the operating cost, and enhances sustainability. The system could be a sustainable solution for urban waste management, even in countries where resources are limited. Also, it can be adjusted to whatever the area in which it is implemented.

While the system demonstrates the feasibility of using a LoRa-based IoT architecture and these techniques, some limitations remain regarding scalability, sensing accuracy, and intelligence. Addressing these limitations opens up multiple directions for future work; for example, optical sensors may fail due to dust and dirt that may accumulate on the sensor. On the other hand, a multi-gateway architecture and IA-assisted decision serve to anticipate the fill level of a bin and optimize waste collection routes automatically.

## Figures and Tables

**Table 1:** Signal strength over the distance.

Transmission No.	Distance (m)	RSSI (dBm)	SNR (dB)	Spreading Factor (SF)	Tx Power (dBm)
1	100	−67	+8.0	SF7	14
2	150	−79	+4.5	SF8	16
3	200	−90	+0.8	SF9	17
4	250	−102	−3.2	SF10	18
5	300	−115	−6.9	SF12	20

**Table 2:** Bin opening records.

No.	Bin ID	Time
1	BIN-01	03:59:06
2	BIN-01	04:30:13
3	BIN-01	04:47:24
4	BIN-01	04:51:31
5	BIN-01	05:01:53
6	BIN-01	05:24:25

**Table 3:** Time interval categories related to user activities.

Interval No.	Time Range (hh:mm)	Typical User Activity	Interaction Frequency
1	00:00–05:00	Late night (low activity)	Very low
2	05:00–08:00	Morning (high activity)	High
3	08:00–17:00	Daytime (moderate activity)	Medium
4	17:00–20:00	Evening (moderate–high activity)	High
5	20:00–24:00	Night (low activity)	Low

**Table 4:** Experimental points for LoRa transmission test.

Test Point	Distance from Gateway (m)	Environment Description
P1	100	Open area, direct line-of-sight
P2	150	Semi-obstructed (trees)
P3	200	Partial obstruction
P4	250	Dense obstruction
P5	300	Maximum coverage range

**Table 5:** Number of transmissions per interval.

Interval	$d_{i}$ (min)	$t_{active\_i}$ (min)	$t_{sleep\_i}$ (min)	${TTC}_{i}$ (min)	${NC}_{i}$
1	300	30.36	0.1667	30.5267	9.827
2	180	3.701	0.1667	3.8677	46.539
3	540	34.83	0.1667	34.9967	15.43
4	180	4.0788	0.1667	4.2455	42.397
5	240	33.66	0.1667	33.8267	7.094

**Table 6:** Total active and sleep duration.

Interval	$T_{active\_i}$ (min)	$T_{sleep\_i}$ (min)
1	1.638	298.347
2	7.758	172.24
3	2.572	537.426
4	7.067	173
5	1.182	238.78

**Table 7:** Comparison of sleep method and idle method result.

Parameter	Sleep Method	Idle Method
Total active time (h)	0.337	0.337
Total sleep/idle time (h)	23.663	23.663
Current (mA)	1.3	10
Total consumption (mAh)	40.03	245.897
Average current (mA)	1.668	10.245
Power consumption (mW)	8.34	51.228

**Table 8:** Power consumption and transmission characteristics at various distance.

Distance (m)	Selected SF	TP (dBm)	RSSI (dBm)	SNR (dB)	t (ms)	Avg. Current (mA)	Energy per Packet (mWh)
100	7	14	−80	6.7	45	95	0.00594
150	8	14	−86	5.1	60	102	0.00850
200	9	18	−92	2.3	90	108	0.01351
250	11	19	−106	0.2	150	115	0.02399
300	12	20	−116	−3.3	220	125	0.03820

**Table 9:** Daily transmission energy with ADRPA.

Distance (m)	$E_{per\_tx}(mWH)$	Daily Energy(mWh)	*Daily* (mAh)
100	0.00594	0.7204	0.1441
150	0.00850	1.0309	0.2062
200	0.01351	1.6386	0.3277
250	0.02399	2.9097	0.5819
300	0.03820	4.6333	0.9266

**Table 10:** ADRPA vs. static (daily savings).

Distance (m)	Static TX (mAh/day)	ADRPA (mAh/day)	Saved (mAh/day)
100	1.8	0.1441	1.6559
150	1.8	0.2062	1.5938
200	1.8	0.3277	1.4723
250	1.8	0.5819	1.1281
300	1.8	0.9266	0.8733

**Table 11:** DSA vs. DSA + ADRPA (daily totals and battery life).

Distance (m)	DSA Daily (mAh)	ADRPA Saved (mAh/day)	DSA + ADRPA Daily (mAh)	*Battery Life* DSA (Days)	*Battery Life* DSA + ADRPA (Days)
100	40.03	1.6559	38.3741	74.94	78.17
150	40.03	1.5938	38.4362	74.94	78.05
200	40.03	1.4723	38.5577	74.94	77.80
250	40.03	1.1281	38.9019	74.94	77.17
300	40.03	0.8733	39.1567	74.94	76.61

## Data Availability

Data are available upon request from the corresponding authors.

## Abbreviations

ADR	Adaptive data rate
ADRPA	Adaptive data rate and power algorithm
BW	Bandwidth
CR	Coding rate
DSA	Dynamic scheduling algorithm
IR	Infrared
MARL	Multi-agent reinforcement learning
MCU	Microcontroller unit
RF	Radio-frequency
RSSI	Received signal strength indicator
RTC	Real-time clock
SF	Spreading factor
SNR	Signal-to-noise ratio
SWB	Smart waste bin
TLA	Three-letter acronym
TP	Transmission power
